# Differential Effects of Climate Warming on the Nectar Secretion of Early- and Late-Flowering Mediterranean Plants

**DOI:** 10.3389/fpls.2018.00874

**Published:** 2018-06-27

**Authors:** Krista Takkis, Thomas Tscheulin, Theodora Petanidou

**Affiliations:** Laboratory of Biogeography and Ecology, Department of Geography, University of the Aegean, Mytilene, Greece

**Keywords:** climate change, floral nectar, Mediterranean plants, nectar resource, optimal temperature, plant–pollinator interactions, seasonal differences

## Abstract

Floral nectar is a vital resource for pollinators, thus having a very important role in ecosystem functioning. Ongoing climate warming could have a negative effect on nectar secretion, particularly in the Mediterranean, where a strong temperature rise is expected. In turn, decreased nectar secretion, together with shifts in flowering phenology can disrupt plant–pollinator interactions and consequently affect the entire ecosystem. Under fully controlled conditions, we tested how temperature influenced nectar secretion (through nectar volume, sugar concentration, sugar content, and number of flowers produced) in six Mediterranean plant species flowering from winter to summer (viz. *Asphodelus ramosus, Ballota acetabulosa, Echium plantagineum, Lavandula stoechas, Rosmarinus officinalis*, and *Teucrium divaricatum*). We compared the changes in nectar secretion under temperatures expected by the end of the century and estimated the effect of climate warming on nectar secretion of plants flowering in different seasons. We found a significant effect of temperature on nectar secretion, with a negative effect of very high temperatures in all species. Optimal temperatures for nectar secretion were similar to the mean temperatures in the recent past (1958–2001) during the respective flowering time of each species. Increasing temperatures, however, will affect differently the early-flowering (blooming in winter and early spring) and late-flowering species (blooming in late spring and early summer). Temperature rise expected by the end of the century will shift the average temperature beyond the optimal range for flower production and the sugar produced per plant in late-flowering species. Therefore, we expect a future decrease in nectar secretion of late-flowering species, which could reduce the amount of nectar resources available for their pollinators. Early-flowering plants will be less affected (optimal temperatures were not significantly different from the future projected temperatures), and may in some cases even benefit from rising temperatures. However, as many earlier studies have found that early-flowering species are more prone to shifts in phenology, the plant–pollinator interactions could instead become affected in a different manner. Consequently, climate warming will likely have a distinctive effect on both plant and pollinator populations and their interactions across different seasons.

## Introduction

Global temperatures show an ever-increasing trend (NOAA, 2018), which is expected to have a considerable effect on numerous species, their interactions and the entire ecosystems (Parmesan and Yohe, 2003; Tylianakis et al., 2008; Traill et al., 2010). In the Mediterranean region, the temperature change by the year 2100 is expected to be particularly strong, with up to 1.5–2.4°C (max 3.0°C) increase in winter and 2.3–3.3°C (max 5.5°C) in summer, in comparison to the second half of the twentieth century (Giorgi and Lionello, 2008; Giannakopoulos et al., 2009; IPCC, 2013). Therefore, climate warming is predicted to have a pronounced effect on Mediterranean ecosystems (Sala et al., 2000; Giorgi, 2006; Malcolm et al., 2006).

Temperature rise can affect plant species and entire communities in multiple ways, by imposing, e.g., phenological shifts (Walther, 2003; Gordo and Sanz, 2009; Bock et al., 2014), physiological temperature stress (Scaven and Rafferty, 2013; Bussotti et al., 2014), and disrupted interactions with mutualists (Memmott et al., 2007). Shifts in phenology in response to climate warming have already been widely recorded in many organism groups across the world (Parmesan, 2006; Cleland et al., 2007; Bertin, 2008; Miller-Rushing and Primack, 2008). In plants, the shifts are usually stronger in early-flowering species and less marked in late-flowering plants (Fitter and Fitter, 2002; Walther et al., 2002; Petanidou et al., 2014).

Ambient temperatures directly affect plant physiology. The optimal range of ambient temperatures for photosynthesis in Mediterranean woody plants under experimental conditions is usually around 25–30° (Flexas et al., 2014), but the optimum can also shift according to season (Medlyn et al., 2002) and be somewhat lower under field conditions (Flexas et al., 2014). Temperatures in the Mediterranean maquis (evergreen-sclerophyllous scrub) reach 35–40°C in summer, but leaf temperature can be even up to 55°C under the same conditions (Larcher, 2000). However, photosynthesis can already start progressively diminishing when leaf temperature is between 35 and 40°C (Larcher, 2000). Altogether, plants in the Mediterranean generally grow under suboptimal temperatures in winter (Larcher, 2000) and close to their optimum (Bussotti et al., 2014) or occasionally even at supra-optimal temperatures in summer (Larcher, 2000; Flexas et al., 2014). However, under future climate warming the optimal temperatures in summer might be exceeded more frequently than before (Bussotti et al., 2014).

Temperature also affects plants indirectly through processes dependent on plant photosynthetic capacity, such as flower and nectar production (Southwick, 1984; Burquez and Corbet, 1998). The effect of elevated temperatures on the number of flowers has been found ambiguous, with both increase and reduction in the number of flowers in different species, or with no change at all (Jakobsen and Kristjánsson, 1994; Liu et al., 2012; Scaven and Rafferty, 2013). A strong heat stress during flowering, however, can cause abortion of buds and open flowers and thus reduce their number (Morrison and Stewart, 2002; Wahid et al., 2007; Bykova et al., 2012). Plants can also produce more flowers without any nectar under temperature stress (Petanidou and Smets, 1996; Takkis et al., 2015). Floral nectar volume is unimodally related to temperature and the optimal temperatures generally correspond well to average ambient temperatures during the flowering season (Jakobsen and Kristjánsson, 1994; Petanidou, 2007). Moderately elevated temperatures may increase nectar secretion (Pacini and Nepi, 2007; Nocentini et al., 2013), but strongly elevated temperatures reduce it (Petanidou and Smets, 1996; Scaven and Rafferty, 2013; Takkis et al., 2015). At the same time, nectar sugar concentration is generally less variable and less affected by temperature than nectar volume (Villarreal and Freeman, 1990; Nocentini et al., 2013; Takkis et al., 2015). Altogether, under elevated temperatures, plant overall nectar secretion could be reduced through a combined negative effect of high temperatures on flower and nectar production.

Combined warming-induced changes in phenology and nectar production can alter plant–pollinator interactions through phenological mismatches and reduced nectar resources available for pollinators (Memmott et al., 2007; Hegland et al., 2009; Petanidou et al., 2014). The most likely reason for mismatches are differences in the cues used by the interaction partners, such as temperature or day length (Hughes, 2000; Bertin, 2008; Doi et al., 2008). Mismatches are more likely to occur among spring than summer species, because of stronger phenological shifts early in the season (Doi et al., 2008; Wolkovich et al., 2012; Fründ et al., 2013). The possible changes in nectar resources are still largely unknown. Consequent changes in the interaction networks can have a negative impact on both plants and pollinators, and cause population declines in both groups (Real and Rathcke, 1991; Hegland et al., 2009; Scaven and Rafferty, 2013). Nevertheless, despite the multiple expected changes, plant–pollinator interaction systems are generally considered to be relatively stable and resilient to climate change (Memmott et al., 2004; Devoto et al., 2007; Petanidou et al., 2014).

In addition to the expected temperature rise, current climate change can also alter precipitation patterns. For the Mediterranean region, different projections generally predict decreased amounts of precipitation (Giorgi and Lionello, 2008; Giannakopoulos et al., 2009; IPCC, 2013). However, the differences in precipitation can be great between adjacent localities—even during the recent hottest years on record, the precipitation patterns in the Mediterranean have been complex, with both less and more than average amounts of rainfall in different places (NOAA, 2018). Furthermore, the magnitude of changes can differ between seasons (IPCC, 2013). Due to the varied patterns of precipitation under climate change (Cook and Wolkovich, 2016), its effect on vegetation in any particular region in the future is expected to be also variable.

In this study, we investigate the effect of temperature on the nectar secretion of six common Mediterranean plant species, flowering from winter to summer. By experimentally provoking nectar and flower production under temperature stress in a climate chamber, we compare the effect of the IPCC-projected temperature rise on the early- and late-flowering species. We expect to find evidence of the negative effect of strongly elevated temperatures on nectar and flower production. We hypothesize that nectar secretion in late-flowering species will be more negatively affected by the predicted climate warming than that of the early-flowering species due to the already very high temperatures characterizing the Mediterranean summer. In the case of different responses of early- and late-flowering species, in combination with the expected phenology changes found in many earlier studies, the effect of climate warming on plant communities, their pollinators, and plant–pollinator interaction networks could have distinctive consequences early and late in the season.

## Materials and methods

### Focal species

**Figure 1:**
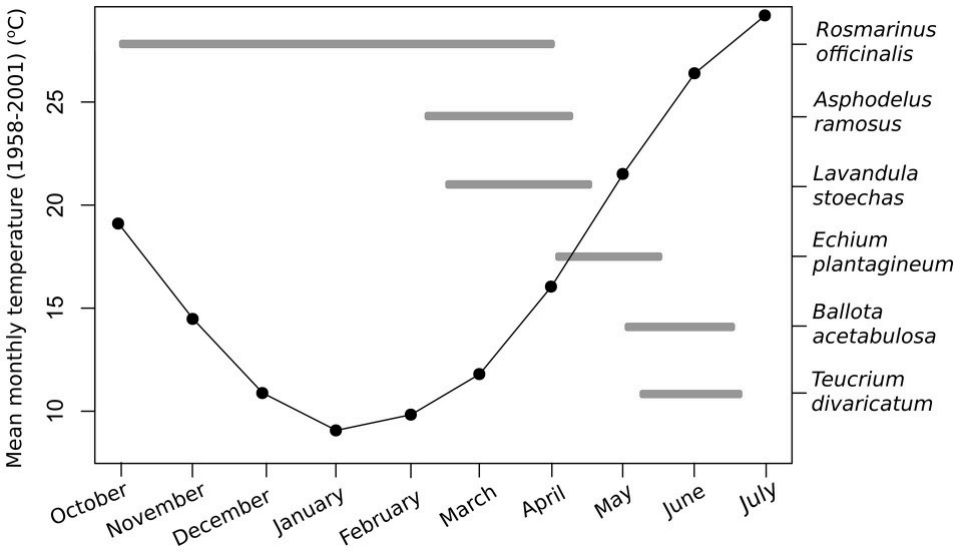
Mean monthly temperatures (1958–2001) and the flowering periods of the six study species in the Aegean region.

We tested the effect of temperature on nectar secretion of six native Mediterranean species that are good nectar producers with flowering periods from winter to summer. The species were (in the order of flowering; Figure 1): *Rosmarinus officinalis* L. (Lamiaceae), *Asphodelus ramosus* L. (Asphodelaceae), *Lavandula stoechas* L. (Lamiaceae), *Echium plantagineum* L. (Boraginaceae), *Ballota acetabulosa* (L.) Benth. (Lamiaceae), and *Teucrium divaricatum* Sieber ex Heldr. (Lamiaceae). All tested species produce relatively large quantities of nectar and are important resources for different pollinators, including honeybees (Herrera, 1988; Petanidou and Smets, 1995; Potts et al., 2006; Keasar et al., 2008; Dauber et al., 2010; Petanidou et al., unpublished data).

Full-grown plants of *R. officinalis* were obtained from a garden center. *Asphodelus ramosus, L. stoechas*, and *T. divaricatum* were collected as entire plants from natural populations on Lesvos Island in October 2013, potted and maintained outdoors until the start of the experiment. *Ballota acetabulosa* and *E. plantagineum* were grown from seeds collected in the wild at the I. & A. Diomedes Botanical Garden of Athens University, Athens, and on Lesvos Island, Greece, respectively. In the case of *B. acetabulosa*, we obtained a subset of seeds collected from *c*. 100 individual plants. The *E. plantagineum* seeds were collected from 30 plants in one population. Plants grown from seeds were germinated in Petri dishes, potted as seedlings and grown outdoors until flowering.

### Experiment design

The temperature response of all species was tested in potted plants in an indoor climate chamber (Walk-in GRW-20 CMP 3/TBLIN, CDR Chryssagis™) during their natural time of flowering in 2014 or 2015. We tested the effect of a wide range of temperatures on plant nectar secretion, aiming to obtain a relatively full response curve for each trait. The selected temperatures were centered around the long-term monthly average temperatures at the time of flowering of each species in the recent past (1958–2001, Elefsis weather station, Athens, Greece). Temperature was increased to at least 3 degrees above the expected temperature maxima according to climate change projections (IPCC, 2013) for that particular season to ensure the stability of the observed trend, or until the flowering finished. We increased the temperature in consistent increments every 3 days. By applying an incremental temperature rise, we allowed for the temperature hardening (acclimatization to higher temperatures) in plants, similarly to natural conditions (Larcher, 2000), which allows us to find the true temperature limitations of these species. Night temperatures were always kept 6°C lower than the day temperatures, simulating natural conditions. The day/night light regime followed approximately the natural diurnal cycles appropriate for the flowering time of each species. Plants were grown under a mixture of plant growth fluorescent lamps (Gro-lux) and low-pressure sodium lamps, with a total light intensity of *c*. 800 μmol m^−2^ s^−1^ (*c*. 43,000 lx) over the waveband 400–700 nm. Relative air humidity was kept constant throughout the experiments, at 60 ± 5% at daytime and 80 ± 5% at night. All plants were watered on Day 1 of each temperature step. For details on the experimental conditions of each species, see Table 1.

**Table 1:** Experimental conditions of the six study species.

	***Rosmarinus officinalis*^a^^,^^b^**	***Asphodelus ramosus*^c^**	***Lavandula stoechas***	***Echium plantagineum*^b^^,^^d^**	***Ballota acetabulosa*^e^**	***Teucrium divaricatum*^e^**
**EXPERIMENTAL GROUP**						
Flowering time (month of peak flowering)	October–April (midpoint January)	March	April	May	June	June
Date	06.−29.01.2015	12.03.−28.03. 2014	30.03.−19.04.2014	05.05.−25.05. 2015	24.05.−17.06. 2014	24.05.−17.06.2014
Day temperatures (°C)	7–34	12–22	16–28	14.5–38.5	20–41	20–41
24 h average temperatures (°C)	3.5–30.5	8.8–18.8	12.8–24.8	12–36	17.5–38.5	17.5–38.5
Temperature increments (°C)	4	2	2	4	3	3
Number of steps	8	6	7	7	8	8
Light/dark (h)	10/14	11/13	11/13	14/10	14/10	14/10
Number of plants	19	12	20	15	15 + 1	15 + 11
**CONTROL GROUP**						
Placement	Outdoors	Climate chamber	–	Outdoors	Outdoors	Outdoors
Date	06.−29.01.2015	03.−24.03. 2015		05.05.−25.05. 2015	24.05.−08.07.2014	24.05.−08.07.2014
Day temperature (°C)	Failed (see text for details)	15		–	–	–
24 h average temperature (°C)		11.8		–	–	–
Number of steps		6		7	15	15
Light/dark (h)		11/13		–	–	–
Number of plants	9	11		6	6	6 + 6

a First increment was 3°C due to technical limitations of the climate chamber.

b *Plants were treated twice during the experiment with the solution of Caster 20SL insecticide to treat a minor parasite infestation*.

c *Due to two general power cuts (lasting several hours but with a prior notice given) the control group experiment had to be stopped twice and the plants were taken outdoors for the time of the blackout to maintain the dark/light regime. The temperature outdoors at the time was similar to that in the chamber. After resuming the experiment, the plants were again given time to adjust to the chamber to ensure equal sampling conditions. As a result, in two cases the time between two measurements was 5 days instead of the usual three. The interruptions did not have any detectable influence on the patterns of flowering and nectar production*.

d *Plants were additionally watered, if necessary, on Day 3 after nectar sampling to retain soil moisture under extremely high temperatures*.

e *Some of the original plants of were replaced when they reached the end of their flowering period, in order to have an equal number of test plants at each temperature step. e.g., one plant was replaced in the case of B. acetabulosa, so that each step would have 15 plants (number of plants: 15 + 1)*.

In addition to the experimental treatments in the climate chamber, we followed control groups of five of the study species (Table 1), to be able to separate the effect of the manipulated temperatures from the natural changes occurring during the flowering period (the effect of time). Plants in a similar flowering stage were randomly divided between the experimental and control group. The controls were in most cases conducted parallel to the experimental treatments and ended when the plants had a comparable number of open flowers as in the experimental group. Only in the case of *A. ramosus*, the control was conducted a year later than the experimental treatment. It was carried out in the climate chamber under controlled conditions with all other settings the same as in the main experiment, but with the temperature kept constant (Table 1).

The controls for *R. officinalis, E. plantagineum, B. acetabulosa*, and *T. divaricatum* plants were conducted simultaneously with the experimental group treatments, but outdoors under naturally varying conditions. The plants were placed in full sunlight under tulle cages to prevent visitation by pollinators. The control data for *R. officinalis* could not be used for the analyses. During the first two sampling periods, there was an unexpected cold spell (near-freezing temperatures) and the plants produced almost no nectar. During the last two sampling periods, the nectar was diluted due to rainfall and was therefore unsuitable for analysis. Consequently, there were too few sampling periods (four out of eight) for reliable use.

### Nectar sampling and the number of flowers

Nectar sampling was conducted uniformly in all species and both in the experimental and control groups. Sampling was performed on Day 3 of every temperature step, starting at 12:30. Nectar was sampled from flowers during their first day of anthesis. To ensure that we only sampled fresh flowers, all flowers were removed on Day 2, 24 h prior to sampling. In the case of *A. ramosus*, the flowers were marked instead of removed, to avoid excessive damage to the plant. Nectar was sampled from three randomly taken flowers per plant using Drummond microcaps® (0.25–10 μl, depending on the size and nectar quantity of the flowers of each plant). Nectar sugar concentration was measured with hand refractometers calibrated for small nectar volumes (Bellingham and Stanley LTD, Tunbridge Wells). Nectar sugar content per flower was calculated based on the measured nectar volume and sugar concentration (volume × concentration × density), with sugar solution density obtained from available tables (page 278 in Dafni et al., 2005). After sampling in Day 3, all new flowers produced during the previous 24 h were counted and removed (or marked). Sugar content per plant was calculated based on the average sugar content per flower and the number of open flowers per plant during Day 3 of each temperature step.

### Climate data

We used the long-term (1958–2001) average monthly temperatures from the Elefsis weather station, Athens, Greece, to compare the optimal temperatures to the average climate conditions in the recent past. The average temperatures during the flowering time of our study species in the region were the following: January 9.2°C, March 11.9°C, April 15.9°C, May 21.3°C, and June 26.2°C (Figure 1). We used the peak flowering time in the nature to make comparisons for each species with past and future temperatures within that month (Petanidou, 1991; Petanidou et al., unpublished data). In the case of *R. officinalis*, we used January for the experiment and comparisons, as it is the approximate mid-point of the plant's long flowering period from autumn to spring (Castro-Díez and Montserrat-Marti, 1998; Keasar et al., 2008).

Future projections for each month considered in the analysis for the Mediterranean region were obtained from the IPCC reports (IPCC, 2007, 2013). We used the projections of the RCP4.5 stabilization scenario (IPCC, 2013), which predicts a 1.5–2.4°C (25th−75th percentiles; max 3.0°C) warming for the winter months (December–February), and a 2.3–3.3°C (max 5.5°C) warming for the summer months (June–August) in the Mediterranean region for the period 2081–2100, compared to the reference period 1986–2005. Since the exact data on spring months were not given for the RCP4.5 scenario, then for this period (March–May) we used the projections of the A1B scenario (IPCC, 2007) that predicts a 2.1–2.7°C (max 3.7°C) warming for the period 2080–2099 compared to the reference period 1980–1999. Both scenarios consider stabilizing greenhouse gas emissions and are comparable in their projections (IPCC, 2007, 2013).

### Data analysis

We tested the effect of temperature on five traits measured per day (Day 3 of each temperature step): (1) nectar volume per flower, (2) nectar sugar concentration per flower, (3) nectar sugar content per flower, (4) nectar sugar content per plant, and (5) the number of flowers per plant. For the first three traits we used average values per plant, i.e., the mean value of the three sampled flowers. When nectar volume in a flower was too small to measure nectar sugar concentration, we inferred this value based on the other flowers sampled from the same plant. This calculation was done in the case of *R. officinalis* for 27/358 flowers, in *L. stoechas* for 43/358 flowers, in *B. acetabulosa* for 11/341 flowers and in *T. divaricatum* for 2/325 flowers.

Nectar volume, sugar content per flower, sugar content per plant and the number of flowers per plant were tested for normality using Shapiro–Wilk test and log-transformed. The sugar concentration data were logit-transformed to remove the constraints of percentage data. All response and explanatory variables were standardized (mean = 0, *SD* = 1) in order to compare the six species. In the case of *T. divaricatum*, we tested for the potential differences between the original and replacement plants (see Table 1) but no significant differences were found (for more details see Takkis et al., 2015).

**Figure 2:**
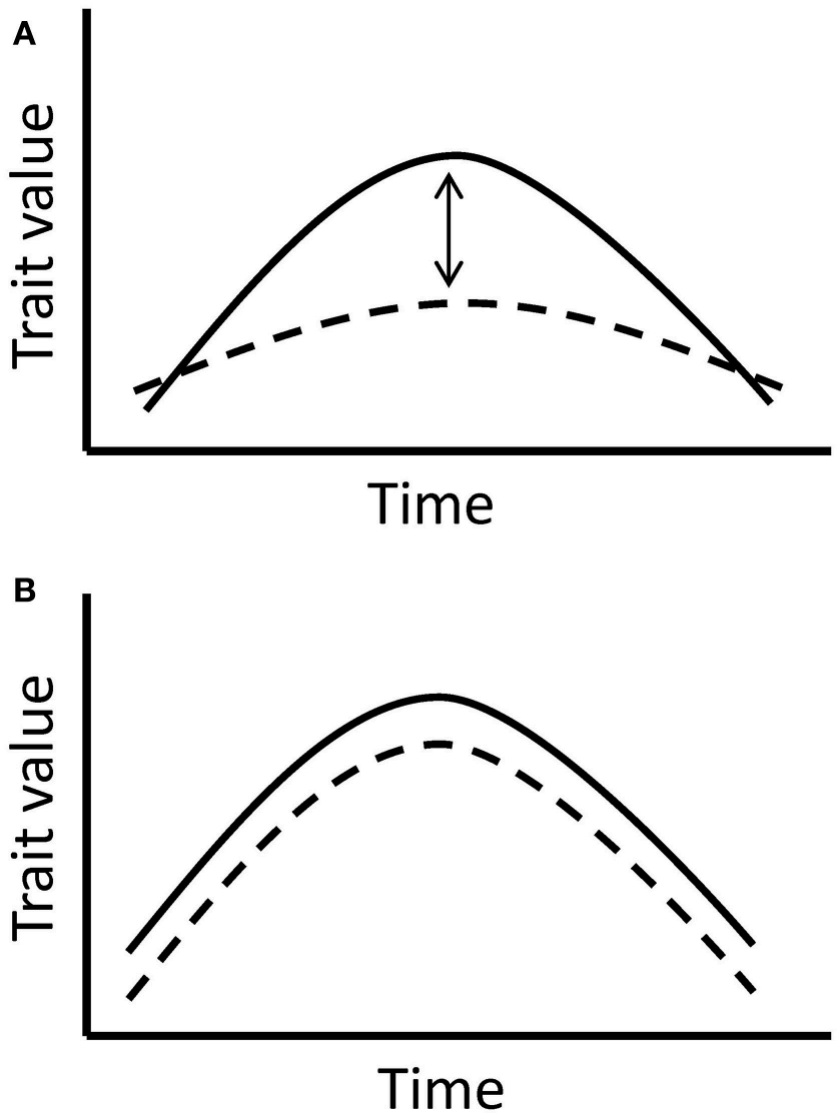
The models compare trait responses to time in the experimental and control group. **(A)** If the interaction between time and treatment group (experimental or control) is significant, it implies a true significant effect of manipulated temperatures indoors, since the effect of time is expected to be similar in all tested plants. **(B)** If the interaction between time and treatment group is non-significant, it indicates the lack of a significant temperature effect in the experimental group and shows a more prevalent effect of time on the trait.

Prior to the main analysis, we examined whether the flower and nectar production trends in the experimental groups were significantly affected by the manipulated temperatures and not caused solely by the natural changes throughout the flowering period. The aim of this analysis was to validate the use of the data only from the experimental group for the following analyses. In order to separate the true effect of manipulated temperatures from the effect of time and natural changes through the flowering period, we compared the experimental and control groups in the four species with reliable control data (Table 1). The comparison is based on the assumption that plants in the experimental and control group respond to time uniformly, but in the experimental group, there is an added effect of elevated temperatures. Hence, a significant interaction between time and treatment in the models would indicate a significant difference between the groups, caused by the elevated temperatures in the experimental group (Figure 2).

For this purpose, we added treatment group (experimental or control) as a binary variable into the linear mixed models (LMM) analysing each trait and used the “time × treatment group” interaction to detect possible differences between the two groups. Separate models were compiled for each of the four species, testing the simple and squared terms of each trait and using plant ID as a random factor in the analyses. Additionally, a combined model for all four species was built, using plant ID nested within the species as a random factor. We tested both simple and quadratic effect of the time and compared which of the models had a better fit based on their AIC values. To be able to compare different trait values among species and different time periods (different length of flowering periods of different species and also outdoors controls sometimes lasted longer than the main experiment), we standardized the parameters (mean = 0, *SD* = 1) when necessary.

For the main analysis, we first divided the six species into two groups–(1) the species flowering in winter and early spring (hereafter early-flowering)–*R. officinalis, A. ramosus*, and *L. stoechas*, flowering between January and April, and (2) the species flowering in late-spring and summer (hereafter late-flowering)–*E. plantagineum, B. acetabulosa*, and *T. divaricatum*, flowering from May to June (Petanidou et al., 1995, 2014). The early- and late-flowering species' phenology often exhibits differential responses to climate warming (Petanidou et al., 1995, 2014; Fitter and Fitter, 2002; Bertin, 2008). Therefore, we could also expect differences between the two groups in other traits, such as nectar production, in response to warming. We tested whether these two groups respond differently to manipulated temperatures, using the interaction of temperature (simple and quadratic effect) and species group (early- or late-flowering) in the linear mixed models (LMM). We used plant ID nested within species as random effects.

**Figure 3:**
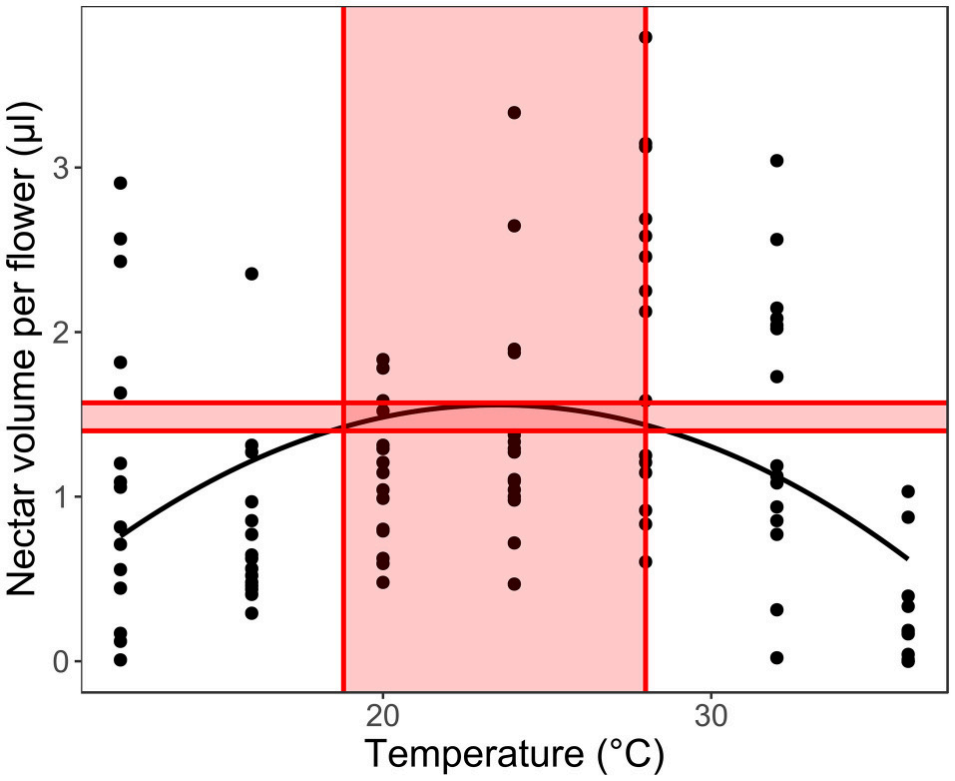
Calculating the optimal temperature range for each trait in each species. We used 5% of the measured trait value range below the calculated optimum as the optimal production range (shaded area between the horizontal lines) to calculate the optimal temperature range for the given trait (shaded temperature values between the vertical lines).

Secondly, we fitted LMM models for each species separately (with plant ID as a random factor), to calculate the optimal temperature range for each trait in each species. We calculated the temperature optimum based on the model maximal values, considering 5% of the highest trait values as the optimal region and the corresponding temperature range as the optimal temperature range for the given trait (Figure 3). In order to understand the response of the early- and late- flowering species, we compared the optimal ranges to the average monthly long-term temperatures in the study region in the recent past (1958–2001, Elefsis weather station, Athens, Greece) and the temperature changes projected for 2100 (IPCC, 2007, 2013) to estimate the species ability to withstand future climate change. We used paired *t*-tests to see if the optimal temperatures of the early- and late-flowering species differ significantly from the past monthly average temperatures and from those predicted for 2100. The tests were conducted for all traits, except for sugar concentration, which in several species had a linear, not unimodal relationship to temperature and therefore did not allow for the optimal range to be calculated in several species.

In addition, we tested the effect of temperature (simple and quadratic effect) on the proportion of empty flowers (flowers producing no nectar; calculation based on the three sampled flowers) in *R. officinalis, L. stoechas*, and *B. acetabulosa*, which had numerous flowers with no nectar. The rest of the species did not have any empty flowers or had very few (in the case of *T. divaricatum*). We used zero-inflated generalized linear mixed models (ZI-GLMM) with negative binomial error distribution for the analysis, with plant ID nested within species as a random factor.

All statistical analyses were conducted in R 3.4.2 (R Core Team, 2017) in the RStudio 1.1.383 environment (R Studio Team, 2016). LMM models were tested using the function *lmer* in the *lme4* package (Bates et al., 2015). Additional *p*-values were calculated with the package *lmerTest* (Kuznetsova et al., 2017). Marginal and conditional coefficients of determination (*R*^2^*m* and *R*^2^*c*) for the LMM models were calculated with the function *r.squaredGLMM* in the package *MuMIn* (Bartoń, 2016). ZI-GLMM models were built with the function *glmmadmb* in the package *glmmADMB* (Fournier et al., 2012; Skaug et al., 2015). Figure S1 was prepared with the function *ggplot* in the *ggplot2* package (Wickham, 2009), using a smoothing function to plot the relationships.

## Results

The results of the control models separating the effect of temperature from that of time based on the comparison with the control groups in four species (Table 2) indicated that nectar volume per flower and sugar content per flower and per plant were significantly affected by manipulated temperatures in the experimental group. This was true in most species separately and in all species combined. Sugar concentration per flower was not affected by temperature in any of the species separately, but showed a significant effect when the species were combined. At the same time, the number of flowers showed a significant response to temperature in the three species separately, but not when the species were combined.

**Table 2:** Difference in the effect of time (simple and quadratic effect, “time” and “time^2^”) on nectar traits and the number of flowers between the experimental and control groups (“group”) in the four species for with reliable control data.

**Trait**	**Interaction terms of the models**	***Asphodelus ramosus***		***Echium plantagineum***		***Ballota acetabulosa***		***Teucrium divaricatum***		**Species combined**	
		***t***	***p***	***t***	***p***	***t***	***p***	***t***	***p***	***t***	***p***
Nectar volume per flower	Time × group	3.119	^**^	−4.900	^***^	−0.757		0.481		−**0.362**	
	Time^2^ × group	0.968		4.881	^***^	3.543	^***^	−2.540	^*^	**3.715**	^***^
Sugar concentration per flower	Time × group	−1.667		1.611		0.310		−0.606		**2.334**	^*^
	Time^2^ × group	−0.501		−0.458		−1.841		1.868			
Sugar content per flower	Time × group	3.529	^**^	−4.988	^***^	0.554		1.058		**2.012**	^*^
	Time^2^ × group	1.417		5.786	^***^	3.880	^**^	−0.997		**4.225**	^***^
Sugar content per plant	Time × group	2.376	^*^	−4.030	^***^	−1.209		−1.041		**0.349**	
	Time^2^ × group	1.008		4.695	^***^	3.007	^**^	−0.066		**2.644**	^**^
Number of flowers per plant	Time × group	0.368		−2.075	^*^	−3.824	^***^	−0.418		−**0.059**	
	Time^2^ × group	−0.823		2.182	^*^	1.027		2.679	^**^	**1.312**	

Only interaction terms are presented here from the model full results and only for the best models (with or without the quadratic effect), according to model AIC values. The analysis with the four species combined is given at the right column in bold.

* 0.05–0.01,

** 0.01–0.001,

*** *<0.001*.

The test indicated that there was a significant effect of manipulated temperatures on the number of flowers and nectar traits (particularly for nectar volume, sugar content per flower and per plant). The effect was relatively consistent across species, indicating therefore that it can be extrapolated with high likelihood to those species where controls were not performed or failed (Table 1). Thus, we conclude that the results of the experimental groups can be used independently to study the effect of temperature across all study species in the following main analyses. In the case of nectar sugar concentration and the number of flowers, the results were more ambiguous and variable across species, and should be used and interpreted with some caution.

In the main LMM analysis, we found that all tested traits were related to temperature either unimodally (for nectar volume, sugar content per flower and per plant, and the number of flowers) or linearly (sugar concentration per flower). However, early- and late-flowering species responded differently to temperature in most traits, except in nectar volume per flower (Table 3).

**Table 3:** Differential dependence of nectar and flower traits on temperature and flowering groups (viz. early- and late-flowering species) in all six species.

	**Nectar volume per flower**	**Sugar concentration per flower**	**Sugar content per flower**	**Sugar content per plant**	**Number of flowers per plant**
Intercept	4.407^***^	−0.179ns	5.343^***^	5.779^***^	4.698^***^
Temperature	−3.788^***^	3.850^***^	−4.989^***^	0.067*ns*	7.523^***^
Temperature^2^	−7.719^***^		0.494^***^	−10.380^***^	−10.831^***^
Flowering group	1.027ns	−0.991ns	−9.667ns	0.640ns	1.326ns
Temperature × flowering group	0.577ns	−6.889^***^	−2.31^*^	−8.322^***^	−15.524^***^
Temperature^2^ × flowering group	−0.737ns		−1.227ns	−0.829ns	0.663ns
*R^2^m*	0.16	0.07	0.28	0.33	0.34
*R^2^c*	0.34	0.29	0.45	0.46	0.56

Given numbers are t-values along with test significance. R^2^m, marginal coefficient of determination, denotes the variation explained by model fixed factors and R^2^c, conditional coefficient of determination, denotes the variation explained by both fixed and random factors together. Only best model results are presented (with or without the quadratic effect), according to model AIC values.

* 0.05–0.01,

*** *<0.001, ns, non-significant*.

**Figure 4:**
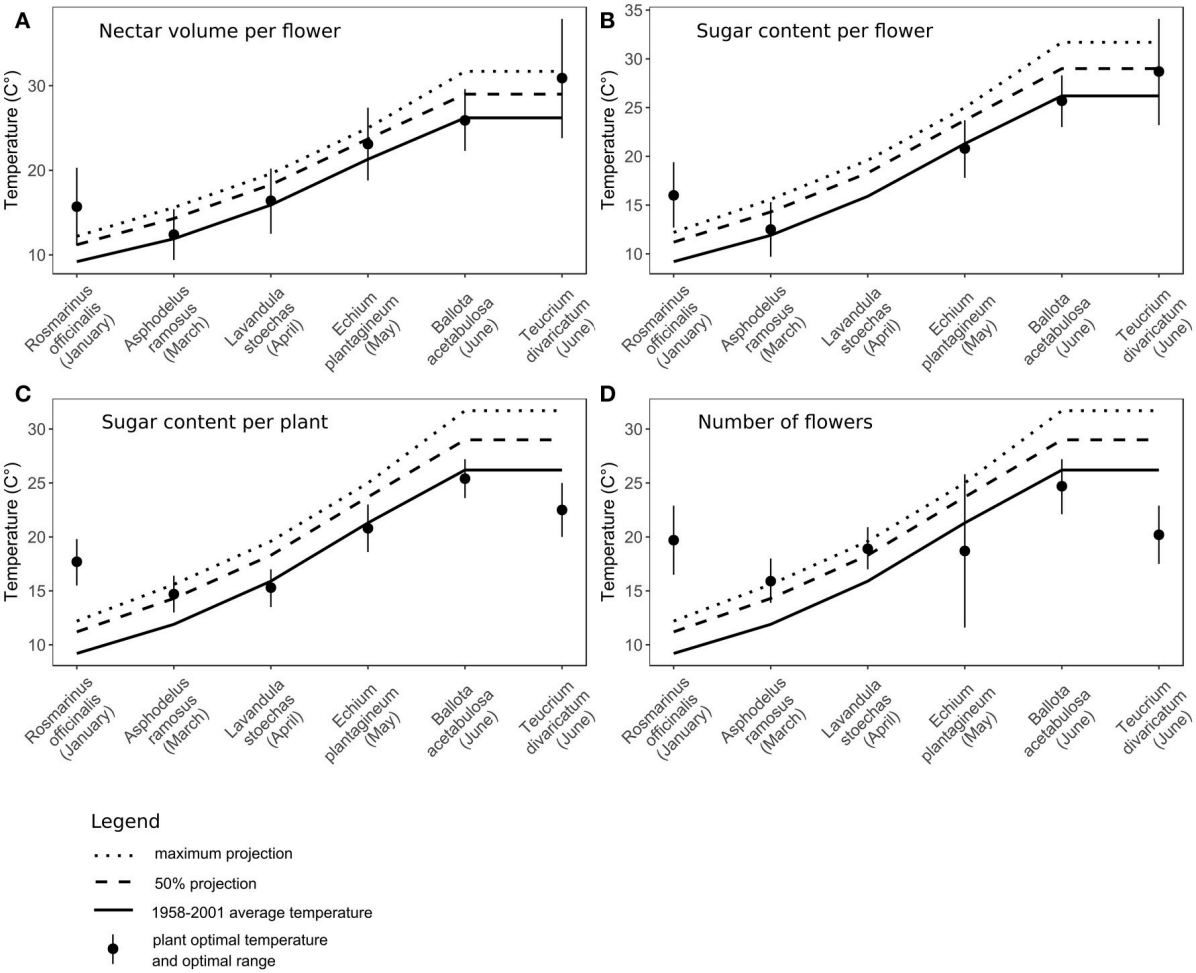
Comparison of optimal temperatures to past monthly average temperatures and future projections by 2100 (IPCC, 2007, 2013) in six species. Flowering month of each species is noted in the parentheses. **(A)** Nectar volume per flower, **(B)** sugar content per flower, **(C)** sugar content per plant, **(D)** the number of flowers per plant. For *Lavandula stoechas*, sugar content per flower (graph B) had a linear relationship to temperature, therefore the optimal temperature could not be calculated.

The optimal temperature ranges showed expected differences among species, but also among different traits within the species (Table 4). Optimal temperatures for nectar volume and flower sugar content followed roughly the monthly average temperatures (Figures 4A–C), whereas the optimal temperature for the number of flowers was more uniform in all species (Figure 4D). Sugar concentration demonstrated the largest variation in trends among species (linear and unimodal, negative, and positive; Table 4).

**Table 4:** Optimal temperatures and optimal ranges for nectar secretion and flower production (24-h average temperatures).

**Species**	**Nectar volume per flower**	**Sugar concentration per flower**	**Sugar content per flower**	**Sugar content per plant**	**Number of flowers per plant**
*Rosmarinus officinalis*	15.7 (11.1–20.3)	–	16.0 (12.7–19.4)	17.7 (15.5–19.8)	19.7 (16.5–22.9)
	⋂^***^	/^***^	⋂^***^	⋂^***^	⋂^***^
*Asphodelus ramosus*	12.4 (9.4–15.4)	–	12.5 (9.7–15.3)	14.7 (13.0–16.4)	15.9 (13.9–18.0)
	⋂^***^	/^***^	⋂^***^	⋂^***^	⋂^***^
*Lavandula stoechas*	16.4 (12.5–20.2)	15.6 (12.5–18.7)	–	15.3 (13.5–17.0)	18.9 (17.0–20.9)
	⋂ns	⋂^**^	^***^	⋂^***^	⋂^***^
*Echium plantagineum*	23.1 (18.8–27.4)	9.4 (0.4–18.4)^a^	20.8 (17.8–23.7)	20.8 (18.6–23.0)	18.7 (11.6–25.8)
	⋂^***^	⋂^***^	⋂^***^	⋂^***^	⋂^***^
*Ballota acetabulosa*	25.9 (22.3–29.6)	26.8 (18.5–35.0)	25.7 (23.0–28.3)	25.4 (23.6–27.2)	24.7 (22.1–27.2)
	⋂^***^	⋃^***^	⋂^***^	⋂^***^	⋂^***^
*Teucrium divaricatum*	30.9 (23.8–37.9)	–	28.7 (23.2–34.1)	22.5 (20.0–25.0)	20.2 (17.5–22.9)
	⋂^**^	\ns	⋂^***^	⋂^***^	⋂^***^

Linear associations are marked with “/” (positive) and (negative); unimodal associations with “⋂” (positive) and “⋃” (negative).

** 0.01–0.001,

*** <0.001, ns, non-significant.

a *The abnormally low, and likely incorrect value is probably caused by the nearly linear relationship of the trait*.

The *t*-tests showed that for all traits the long-term average temperatures in the recent past are comparable to the optimal temperatures for nectar volume per flower, sugar content per flower and per plant and the number of flowers per plant (Table 5, Supplementary Material). In the case of nectar volume per flower, the future projected temperatures are also not significantly different from the optimal temperatures. In the case of early-flowering species, non-significant results were also found for all other traits, whereas the tests were significant or marginally significant for late-flowering species, indicating stronger differences between the optimal and projected temperatures in the future in this plant group (Figure 4, Table 5).

**Table 5:** Comparison of the optimal temperatures within a species group (early- and late-flowering) to the average monthly temperatures in the recent past and future projections for the year 2100 (IPCC, 2013) in the month of flowering of each species (results of paired *t*-tests).

**Trait**	**Early-flowering species**			**Late-flowering species**		
	***d.f*.**	***t***	***p***	***d.f*.**	***t***	***p***
**NECTAR VOLUME PER FLOWER**						
1958–2001 monthly average	2	1.250	ns	2	1.426	ns
50% projections	2	0.109	ns	2	−0.416	ns
75% projections	2	−0.048	ns	2	−0.715	ns
Maximal projections	2	−0.433	ns	2	−1.868	ns
**SUGAR CONTENT PER FLOWER**						
1958–2001 monthly average	1	1.194	ns	2	0.500	ns
50% projections	1	0.455	ns	2	−2.304	ns
75% projections	1	0.354	ns	2	−2.837	ns
Maximal projections	1	0.102	ns	2	−5.047	^*^
**SUGAR CONTENT PER PLANT**						
1958–2001 monthly average	2	1.344	ns	2	−1.633	ns
50% projections	2	0.468	ns	2	−3.932	.
75% projections	2	0.352	ns	2	−4.158	.
Maximal projections	2	0.035	ns	2	−4.530	^*^
**NUMBER OF FLOWERS PER PLANT**						
1958–2001 monthly average	2	2.481	ns	2	−2.486	ns
50% projections	2	1.436	ns	2	−4.316	^*^
75% projections	2	1.319	ns	2	−4.541	^*^
Maximal projections	2	0.916	ns	2	−5.074	^*^

.*0.1–0.05,*

* *0.05–0.01, ns, non-significant*.

The proportion of empty flowers in *R. officinalis, L. stoechas*, and *B. acetabulosa* had a negative unimodal response to temperature, indicating a considerably higher production of empty flowers at higher temperatures, but also a slightly higher occurrence at the lowest temperatures (Table 6).

**Table 6:** The percentage of empty flowers in *Rosmarinus officinalis, Lavandula stoechas* and *Ballota acetabulosa* in relation to temperature.

	**Estimate**	***SE***	***z***	***p***
Intercept	3.843	0.092	41.81	^***^
Temperature	0.087	0.033	2.61	^**^
Temperature^2^	0.133	0.040	3.34	^***^

Model standard errors (SE) and z-values are presented in the table.

** 0.01–0.001,

*** *<0.001*.

## Discussion

We found that the progressing climate warming could alter nectar and flower production in different Mediterranean species by the end of this century. The optimal temperatures for nectar secretion in most traits were close to the long-term average temperatures in the recent past (Figure 4, Table 5), confirming the plants' adaptation to past climate conditions (Jakobsen and Kristjánsson, 1994; Bussotti et al., 2014). In the case of *R. officinalis*, the optima were higher than the past average temperatures in January (the mid-point of its flowering period), indicating the species' adaptation to a wider range of temperatures, which matches its long flowering period from autumn to spring. Extremely high experimental temperatures reduced nectar secretion in all species through reduced volumes, sugar content, number of flowers, and a greater proportion of empty flowers in some species, corresponding to earlier studies in different plant species (Petanidou and Smets, 1996; Keasar et al., 2008; Scaven and Rafferty, 2013). Admittedly, within the frame of the future temperature rise by 2100 (IPCC, 2013), the actual effect of warming on nectar secretion will likely be less pronounced and differing between seasons.

The effect of expected warming was significantly different on early- and late-flowering species nectar secretion. Both early- and late-flowering species responded similarly regarding nectar volume per flower, which was not compromised by the rising temperatures projected for 2100 (Table 4). However, nectar volume could be more susceptible to possible additional reduction in soil humidity coinciding with rising temperatures than to temperature rise *per se* (Villarreal and Freeman, 1990; Petanidou, 2007; IPCC, 2013). In the rest of the traits, the early- and late-flowering species differed in their responses (Tables 2, 4). Sugar concentration showed a positive response to elevated temperatures in early-flowering species and negative response in the case of late-flowering species. However, the individual responses were very variable between species (Table 4) and the pure effect of temperature uncoupled from time was somewhat questionable (Table 3), therefore the effect of concentration changes within the frame of the future warming is difficult to interpret. In the case of sugar content per flower and per plant, and the number of flowers, for the early-flowering species the optimal temperatures will not be significantly surpassed under future warming. In the late-flowering species, however, nectar sugar content per flower and per plant could be marginally affected by the rising temperatures by the end of the century. Elevated temperatures could compromise nectar sugar content in late-flowering species at least occasionally during the hotter parts of the day or during heat wave events (Larcher, 2000; Bussotti et al., 2014), which are predicted to become more frequent in the future (Giannakopoulos et al., 2009; Rahmstorf and Coumou, 2012; IPCC, 2013). The greatest negative impact of elevated temperatures on late-flowering species will probably be through the number of flowers, at least in multi-inflorescence species, such as most of our study species (except for *A. ramosus*), which reduce their number of flowers under heat stress (Table 2; Liu et al., 2012). Reduced number of flowers can in turn strongly affect the whole plant's nectar secretion and thus the available resources for pollinators.

Seasonal differences of the effect of climate warming on nectar secretion could be expected in the future. Early-flowering species' nectar secretion might benefit from the temperature rise, whereas late-flowering species could be moderately disadvantaged. Some species flowering very early in the year could encounter temperatures closer to their optimum than the past ones (Llorens et al., 2003) and produce higher amounts of nectar and sugar. For other early-flowering species, the optimal temperatures might be surpassed to some degree, but not significantly (Figure 4, Table 5). Also, the phenology of early-flowering species is found to be relatively flexible, so under warming they can shift their phenology to remain within their optimal temperature range (Post and Stenseth, 1999; Fitter and Fitter, 2002; Walther et al., 2002). At the same time, the conditions can become increasingly harder for species flowering toward summer. Temperatures in the Mediterranean are expected to rise in the future more rapidly in summer than in any other season (Giorgi and Lionello, 2008; IPCC, 2013) and can surpass the optimal temperatures for nectar and sugar production. Mediterranean plants are generally well adapted to high temperatures and summer drought (Gratani and Varone, 2004; Petanidou, 2007; Miranda et al., 2011; Nuru et al., 2012). However, late-flowering species are already close to or beyond the optimal temperatures for photosynthesis (Larcher, 2000; Bussotti et al., 2014; Flexas et al., 2014), which determines the resources available for flower and nectar production (Southwick, 1984; Burquez and Corbet, 1998; Pacini et al., 2003). Therefore, any increase in temperature can decrease the functioning of late-flowering species more easily compared to early-flowering ones. The optimal temperatures for flower production in summer are already now slightly exceeded (although non-significantly) by the monthly average temperatures and will be significantly surpassed in the future (Figure 4, Table 5), threatening plants with decreased flower production and reduced overall nectar production.

The potential effect of altered resource availability on pollinators can likewise be different early- and late in the season. Early-flying species would probably not be directly affected by reduced quantity or quality of nectar. However, they could be faced with plant phenology shifts often found in early-flowering species, which can indirectly alter the amount of nectar resources available (Bertin, 2008; Wolkovich et al., 2012; Petanidou et al., 2014). Early-flowering plants, at the same time, can lose a number of pollinators due to phenology mismatches and receive lower pollination service as a result (Petanidou et al., 2014). Pollinators flying later in season will probably not be affected by plant phenology shifts (Bertin, 2008; Petanidou et al., 2014), but might need to cope with moderately reduced amounts of nectar, at least during heatwaves or hotter periods of the day. Altogether, altered plant–pollinator interactions could have distinctive effects on both plant and pollinator populations in different seasons.

It is important to note that the effect of climate change on plants is not limited to temperature, but also includes other climatic variables, such as precipitation (Giannakopoulos et al., 2009; Coumou and Rahmstorf, 2012; IPCC, 2013; Petanidou et al., 2018). Altered rainfall patterns can either enhance or alleviate the effects of elevated temperatures (Bussotti et al., 2014; Cook and Wolkovich, 2016). Changes in precipitation under climate warming are highly variable and dependent on local conditions (NOAA, 2018). Therefore, in this study we limited our work only on testing the effect of temperature rise on plants, to discern the singular effect of temperature rise on plants, uncoupled from potential precipitation changes. We certainly acknowledge the possible additional effect of changed rainfall patterns on plants and their nectar secretion (Villarreal and Freeman, 1990; Carroll et al., 2001; Petanidou, 2007), which affects the overall impact of climate change on plant nectar production and plant–pollinator interactions (Petanidou et al., 2018).

It is possible that during the next century, plants will be able to adapt to some degree to climate warming (Parmesan, 2006). Plants are able to adjust their physiology (such as photosynthetic optima) through the annual temperature changes (Medlyn et al., 2002) or elevational differences (Fryer and Ledig, 1972). In fact, both plastic (Nicotra et al., 2010) and rapid evolutionary responses to climate change have been recorded in plants (Jump and Peñuelas, 2005). However, it is hard to predict how much the adaptational shifts could mitigate the negative effects of warming on flower and nectar production. Conditions in the Mediterranean region in summer are already very difficult for plants (Larcher, 2000; Bussotti et al., 2014) and the potential for adaptation to even harsher conditions, on a relatively short time-scale, might be limited.

We conclude that future temperature rise could have a negative effect on the nectar and flower production of Mediterranean plant species, particularly on the late-flowering species blooming from late spring to summer. The effect of climate warming on plant species and plant–pollinator interactions could be markedly different between seasons and these differences need be taken into account when estimating the overall effects of climate change. Having a more thorough knowledge of the effect of temperature rise on different plant traits, various species and the differences through seasons is essential to comprehend the effect of warming on whole communities and ecosystems through altered interaction networks. Our results on the effect of temperature on the nectar secretion of different plants give a good basis for further studies on (i) the effect of different climatic factors (such as precipitation changes), (ii) effects on more detailed plant–pollinator interaction networks, and (iii) for tests under natural conditions, which could further advance our knowledge of the impact of climate change on ecosystem functioning.

## Author contributions

TP conceived the idea and found funding for the study, and devised the experiments with the contribution by TT. KT conducted the experiments and analyzed the data. All authors contributed to the writing of the manuscript, which was led by KT.

### Conflict of interest statement

The authors declare that the research was conducted in the absence of any commercial or financial relationships that could be construed as a potential conflict of interest.
