# Supplementary material for: Differential Effects of Climate Warming on the Nectar Secretion of Early- and Late-Flowering Mediterranean Plants
Source: Front Plant Sci. 2018 Jun 27;9:874. doi: 10.3389/fpls.2018.00874 (PMC6030359; doi:10.3389/fpls.2018.00874)

Supplementary Material

Differential effects of climate warming on the nectar secretion of early- and late-flowering Mediterranean plants

Krista Takkis[*], Thomas Tscheulin, Theodora Petanidou

*** Correspondence:** Krista Takkis: krista.takkis@gmail.com

Figure S1. Nectar and flower traits in relation to temperature in early- (filled circles and solid lines) and late-flowering species (empty circles and dotted lines). Variables on both axes are standardized (mean = 0, *SD* = 1). Grey bands around the smoothing lines denote the 95% confidence intervals. Mean tested temperature (zero on the x-axis) was in all cases close to the average temperature in the species' flowering season.

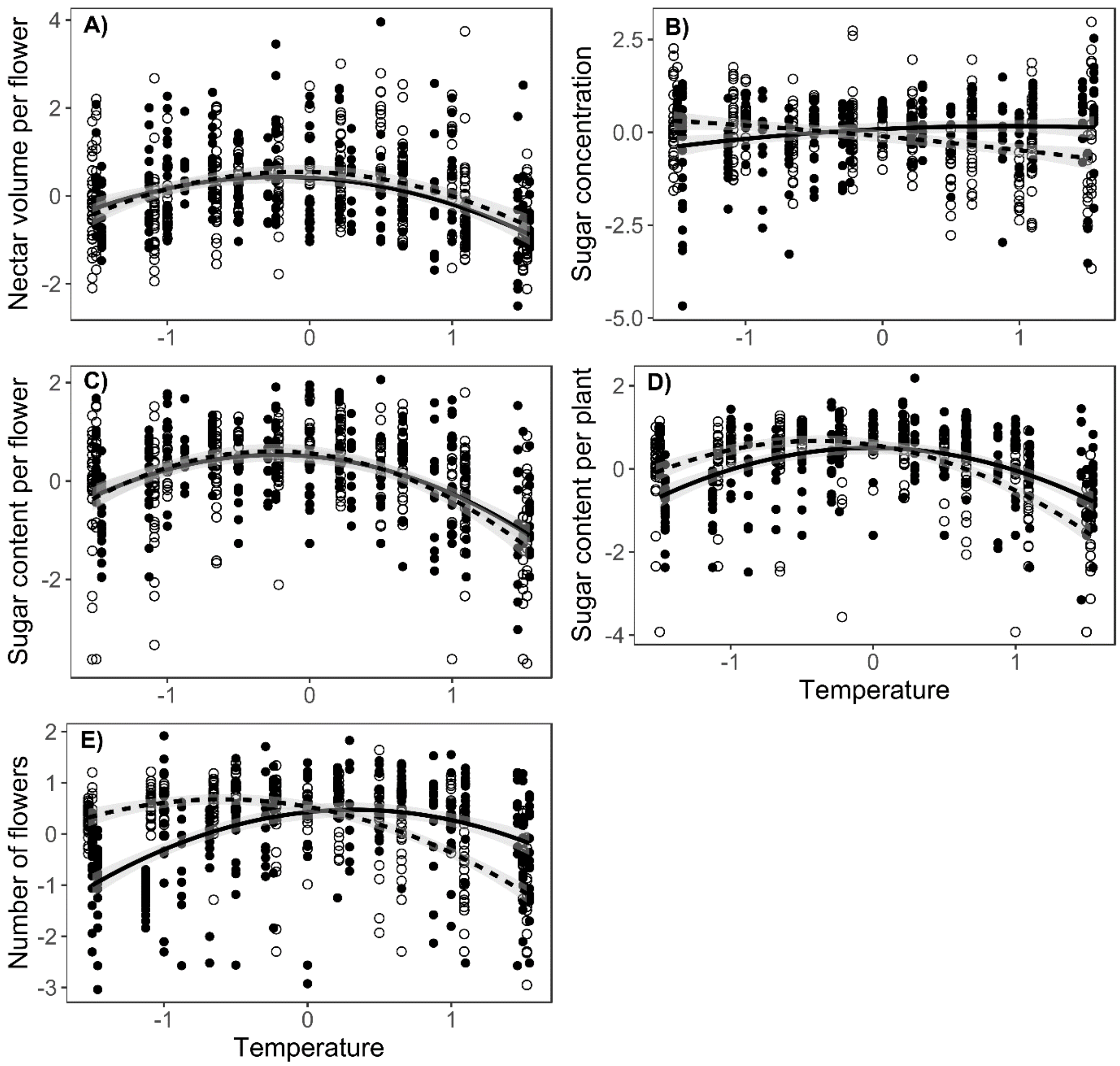

Supplement: Supplementary file 1 [file Image_1.PDF]
